# Rural-Urban Differences of Dietary Patterns, Overweight, and Bone Mineral Status in Chinese Students

**DOI:** 10.3390/nu8090537

**Published:** 2016-09-06

**Authors:** Yang Yang, Xiao-Mei Hu, Tian-Jiao Chen, Ming-Jie Bai

**Affiliations:** 1 Institute of Child and Adolescent Health, School of Public Health, Health Science Center, Peking University, 38 Xueyuan Road, Haidian District, Beijing 100191, China; yyangsunseason@yeah.net; 2 Affiliated Hospital of Zunyi Medical College, Zunyi 563000, China; gzhxm7661@163.com; 3 Medical School, Anhui University of Science and Technology, Huainan 232001, China

**Keywords:** dietary pattern, overweight/obesity, central adiposity, risk of bone fracture, adolescent students, rural-urban disparity

## Abstract

China is an urban and rural social model country. In the past three decades, the developing speed of rural areas has been much slower than urban areas, which may lead to the differences in dietary patterns. This study aimed to investigate the disparities of dietary structures from urban and rural children, and to analyze the effects of different dietary patterns on their adverse outcome. Among 1590 students, aged 11 years to 17 years, from primary and middle schools, a cross-sectional study was conducted. There were three dietary patterns recognized: Westernization structure, meat diet structure, and Western and Chinese structure. Compared with rural students, more urban students were in the highest categories of the whole dietary patterns (*p* < 0.001). Overweight/obesity and central adiposity were more prevailing among urban students, while rural students had a more prevailing risk of bone fracture (*p* < 0.05). Through the adjustment for all confounding factors, the Westernization structure could increase the risk of overweight/obesity and central adiposity, the meat structure could increase the risk of elevated blood pressure/hypertension, while the risk of low bone mineral quality could be reduced by the Chinese and Western structure. In conclusion, a rural-urban disparity in dietary patterns was found in our study, and different dietary patterns were associated with the risk of some adverse outcomes. Therefore, there were different prevalences of the adverse outcomes between rural and urban students.

## 1. Introduction

As the largest developing country worldwide, over 60% of China’s population live in rural regions. China is a typical urban and rural social structure country. In the past three decades, the developing speed of rural areas has been much slower than urban areas [1], and economic development disparities may result in the differences of food supply and consumption patterns, thus influencing dietary patterns and the status of physical constitution [2]. Some research has found that the changes of dietary patterns were greatly related with the disparities in metabolic syndrome, bone mineral status, and overweight/obesity between urban and rural regions [3,4,5,6]. According to the 2002 China National Nutrition and Health Survey (CNNHS) the data showed that, compared with urban teenagers, both boys and girls, rural teenagers were 4–5 cm shorter at the same age from three to eighteen years old [7]. Meanwhile, central adiposity, overweight/obesity, as well as diabetes and hypertension, are extremely prevalent in developed coastal or urban regions than underdeveloped and remote rural regions [8].

The disparities in dietary patterns make contributions to the different nutrition condition and body composition in urban and rural regions. Understanding the disparities in dietary patterns between urban and rural regions can contribute to a better evaluation of health demands. Few studies were conducted to study the differences of dietary patterns between urban and rural regions. The goal of this research was to investigate the differences in dietary patterns between urban and rural regions and adverse outcomes, such as central adiposity, overweight/obesity, elevated blood pressure/hypertension, and the risk of bone fracture among Chinese students.

## 2. Materials and Methods

### 2.1. Participants

Two primary schools and two junior high schools, respectively, from Suqian, Jiangsu and Xingxiang, Henan, were randomly allocated, using the cluster sampling approach. Students from grade four to grade six of two primary schools and all of those from two junior high schools were included. The survey was carried out in May 2014. Participants of each class were instructed to complete the respective questionnaires at a same time. Assessments of anthropometry, blood pressure, and speed of sound, a predictor of risk of fracture, were scheduled at each school. In total, 2214 students were selected to fill out the questionnaires, among which 1853 agreed to complete the measurement of indicators. 263 of these students were excluded from the study owing to incompletion of the questionnaire. After the exclusion, we included 1590 students aged 11 years to 17 years. We divided subjects into an urban group (who choose county and above the county-level cities) and a rural group (villages and towns) according to the question that “Where is the location of your home”. Some prizes were offered to students as compensation for their participation in the evaluation. Written informed consent was obtained from the students and their parents before they participated in this study. This study was approved by the Medical Ethics Committee of Affiliated Hospital of Zunyi Medical College.

### 2.2. Assessment of Dietary Intake

A food-frequency questionnaire was used to evaluate the dietary intake, which was modified based on the previous questionnaire in accordance with the dietary characteristics of target group [9]. Twenty-four food groups are included in the modified questionnaire that Chinese people frequently consume as shown in Table 1. With each item standing for a food group, all participants were asked how often (daily, weekly, monthly, annually or never) they consumed each food group. Validity and reliability of the questionnaire was evaluated. A pilot survey was used to validate the questionnaire among 30 students of a small class through comparing with three 24-h dietary recalls before the study, and the range of the correlation coefficients between the FFQ (Food frequency questionnaire) and averages from 24-h dietary recalls was from 0.31 and 0.69 for major food groups. The test of reliability was conducted to finish the FFQ questionnaire within the same population, and one month later, the second FFQ questionnaire survey was conducted. Comparing the two FFQs, it was found that the range of Pearson correlation coefficients was from 0.35 to 0.70 for main food groups. Therefore, the data showed that the FFQ ensured the validity and reliability of dietary intakes measurements.

The whole questionnaire was administered by an educated dietitian. Each participant was asked the frequency of his consumption of every food group during the last one year. There were three categories of frequency consumption, daily, which represented core food; weekly, which represented secondary core food; and monthly, which represented peripheral food. All categories were scored by “never” = 0; “1–3 times” = 1; “4–5 times” = 2; “6–8 times” = 3; and “over 8 times” = 4. The FFQ focused only on the frequency of each food item, and information on the portion size was not included.

### 2.3. Assessment of Anthropometric Measures

A Seca (HW-700, Kai Yuan Corporation, Zheng Zhou, China) scale was used to measure weight which was recorded to the closest 0.1 kg. A Seca stadiometer was applied to measure height. All subjects were required to keep in a standing position and their shoulders stayed in normal position. Weight (kg) divided by square of height (m) was used to show body mass index (BMI). Based on the research of body mass index reference norm for screening overweight and obesity in Chinese children and adolescents conducted by the Working Group on Obesity in China [10] and the screening standard for malnutrition of school-age children and adolescents published by National Health and Family Planning Commission of the People’s Republic of China, the values of BMIs for each age and sex can be divided into categories: obesity/overweight, normal weight, and wasting.

Waist circumference (WC) was measured to the narrowest level between the lowest rib and the iliac crest, and hip circumference was measured at the maximum level over light clothing with the use of an outstretched tape measure. Measurements were recorded to the nearest 0.1 cm, and the waist-to-hip ratio (WHR) was calculated. With reference to the norms of waist circumference [11], when WC was equal or over ninetieth percentile for children with same age and sex, it was defined as central adiposity in this study.

### 2.4. Assessment of Other Variables

Before blood pressure was measured, participants were required to have a 15-min rest. Blood pressure of seated participants was measured twice by a licensed physician with a standard mercury sphygmomanometer, and the mean value of the two results was obtained as their blood pressure. The occurrence of the first sound referred to systolic blood pressure (Korotkoff phase 1), while the vanishing of the sound referred to diastolic blood pressure (Korotkoff phase 5) when the cuff deflated with the mercury column at the attenuation ratio of 2 to 3 mm/s. According to the blood pressure reference standards, diastolic blood pressure (DBP) and/or systolic blood pressure (SBP) of over the ninetieth percentile for children with the same age and sex meant elevated blood pressure. DBP and/or SBP of over the ninety-fifth percentile for children with the same age and sex meant hypertension [12].

An ultrasound bone densitometer was used to assess risk of fracture by measuring speed of sound (SOS, m/s), an indicator of bone quality, on the right calcaneus (CM-200, Furuno Electric Corporation, Hyogo Nishinomiya, Japan). The right foot was disinfected on both sides, and the speed of sound (SOS) was measured twice. SOS (m/s) results were presented in *Z*-scores, which are matched for age and sex, allowing comparisons to peers [13].

A Chinese version of the International Physical Activity Questionnaire (IPAQ-C) was used as an instrument for physical activity measurement in this population, and the reliability and validity of the questionnaire has been examined [14]. With the application of the IPAQ-C, physical activity of participants was measured, which included three grades: low-intensity exercise, medium-intensity exercise, and strenuous exercise, respectively. Participants were asked how much time they exercised every week with three categories: never, less than 3 h/week, and 3 h/week or more. Additional covariate information regarding age, sex, passive smoking, economic status, education of father/mother, place of meals, and nutritional supplements was obtained by using questionnaires.

### 2.5. Statistical Analysis

The factor analysis with varimax rotation was used to recognize major dietary structures according to 24 food groups. Main dietary structures referred to the factors with Eigen values of over 1.5. Factor loadings represent the correlation coefficients between dietary structures and personal food groups. The proportion of variance explained by each factor was calculated by dividing the sum of the squares of the respective factor loadings by the number of variables. The sum of intakes of every food group was used to measure the scores of factors for every pattern and for each person, which was weighted by the factor loadings [15].

The associations between categorically demographic variables and dietary patterns were assessed using χ^2^ analysis. *T*-test was used to compare continuous variables (mean ± SD). Multivariable logistic regression was applied to recognize the risk of overweight/obesity, central adiposity, abnormal blood pressure, and low bone quality for each main dietary structure. Possible confounding factors were controlled in the patterns of regression. The first tertile of dietary model values was regarded as a reference in term of all analyses. SPSS 16.0 (SPSS, Inc., Chicago, IL, USA) was applied and *p* < 0.05 was vitally important in statistics.

## 3. Results

A total of 1590 students aged 11 years to 17 years (mean age, urban 14.98 ± 1.16 years, rural 15.18 ± 1.27, respectively) were investigated. Table 2 showed the distribution of sociodemographic features of urban and rural students. The differences of mean age and sex distribution were not significant between urban and rural students. Students from urban schools were more likely to be from wealthy families, have parents with higher education level, eat at home, and take nutritional supplements accounted for a higher proportion (*p* < 0.001), while those from rural schools were more likely to be from poor families, have parents with lower education level, eat at canteen, and take nutritional supplements accounted for a lower proportion (*p* < 0.001).When compared with rural students, urban students had less physically activity (*p* < 0.001).

With the use of questionnaires and factor analysis, three dietary patterns were chosen from 24 food groups as shown in Table 1. Table 1 also presents the factor-loading matrices. The three dietary patterns were, respectively, Chinese and Western structure, which was identified with high consumption of rice and rice products, flour and flour products, whole grains, fresh vegetables, fresh fruit, poultry, eggs, freshwater fishes and shrimps, deep-sea fish, milk and dairy products, beans and bean products, nuts, snacks, sugar, and barbecue; Westernization structure, which was identified with high intakes of rice and rice products, flour and flour products, fresh fruit, fat meat, hamburgers and fried foods, processed products, snacks, coke, sprite, coffee, ice cream, instant noodles, and barbecue; and meat diet structure, which was identified with intakes of fat meat, beef and mutton, poultry, animal liver, freshwater fishes and shrimps and deep-sea fish, as well as processed products. Totally, in the dietary intake, 65.6% of the variations were illustrated by these dietary structures.

According to Table 3, compared with rural students, more urban students were in the highest categories of the whole dietary patterns (*p* < 0.001). In other words, urban students had a tendency to follow the Chinese and Western pattern, Westernization structure, and meat dietary model more than rural students.

As was shown in Table 4, the differences of basic characteristics between urban and rural regions were significant between boys and girls. Compared with students from rural schools, those from urban schools had a higher level of weight, waist circumference, hip circumference, and SOS both for boys and girls (*p* < 0.05). Consequently, urban students had a higher proportion of overweight/obesity and central adiposity (*p* < 0.05). However, rural students had a lower proportion of low bone quality both for boys and girls (*p* < 0.01).

In multiple logistic regression analyses, all dietary patterns were regarded as exposure variables and categorical overweight/obesity, central adiposity, elevated blood pressure/hypertension, and low bone quality as outcome variables. The results in Table 5 indicated that the higher values in the Westernization pattern had relationship with higher risk of overweight/obesity (T3 vs. T1: OR = 1.925, 95% CI: 1.299–2.852) and central adiposity (T3 vs. T1: OR = 5.511, 95% CI: 2.222–13.667), which were strengthened by control for factors, such as sex, passive smoking, drinking, calcium supplements, bone mineral density (BMD), BMI, and physical activity in model 3. After adjusting for all confounders in model 3, elevated blood pressure/hypertension has positive association with the values in the meat dietary pattern (T3 vs. T1: OR = 1.576, 95% CI: 1.156–2.147). The Chinese and Western pattern was associated with decreased risk for low bone quality in model 3 (T3 vs. T1: OR = 0.421, 95% CI: 0.289–0.559).

## 4. Discussion

We identified three dietary patterns in Chinese primary and middle school students. Obvious differences of dietary patterns between urban and rural regions were investigated in terms of Chinese students in this research. Different dietary patterns were associated with the risk of some adverse outcomes; for example, overweight/obesity, central adiposity, elevated blood pressure/hypertension, and low bone quality. Based on the disparities of dietary patterns between urban and rural regions, the differences of basic characteristics can be illustrated. Actually, there were few studies researching the disparities of dietary patterns in China. To the best of our knowledge, this is the first research on the investigation of disparities of dietary patterns and their relationship with overweight/obesity, central adiposity, and bone quality among Chinese urban and rural children and adolescent students.

China is the largest developing country with a large rural population who differs from urban residents with regard to lifestyle, culture, socioeconomic status, and diet [16]. Thus, their dietary patterns are significantly different from those of their urban peers. The food system can be divided into two aspects in China: residents in large cities, such as Beijing, Shanghai, and Guangzhou have changed into a more urbanized, Westernized lifestyle, while residents in rural regions are limited in food diversity. In this research, more urban students are in the highest tertile of Chinese and Western, Westernization, and meat diet dietary models, while more rural students are in the lowest tertiles of the three dietary structures.

In our study, it was found that rural students had lower bone quality than urban students, which indicated a lower BMD. In contrast, previous studies conducted in Western countries showed that rural populations instead of urban ones had a higher BMD. Based on a Swedish study of 246 participants aged 15–16 years, Martin et al. [17] reported that the total body BMD and lumbar spine BMD were higher in rural adolescents compared with urban adolescents even after adjustment for physical activity and BMI. The reason for the inconsistency between Chinese students and Western students is still unclear. Research on Chinese immigrants (mean age, 35 years) to the west is possibly helpful. Wang et al. [18] found that Chinese population who had lived in Denmark for more than 12 years had a similar BMD to that of Danish population, while among those who immigrated to Denmark less than 12 years ago, a significantly lower BMD was found. The reason was due to a more westernized lifestyle and dietary pattern. In fact, Chinese dietary patterns have been changing in the past two decades. There is a finding in our study that rural students were less likely to have a Chinese and Western pattern and this pattern was, indeed, associated with a decreased risk of lower BMD. High intakes of milk and milk products could increase their bone mineral density [19,20]. Moreover, we also found that the physical activity of urban children was significantly lower than rural children. Physical activity is one of the important environmental factors to improve bone mineral density and, as a result, reduces the risk of fracture. Plenty of research has shown that physical activity has a positive effect on bone growth [21,22], and the increase of bone mass of children with high physical activity was significantly higher than children with low physical activity [23,24]. However, it was found that urban students had characteristics of a higher calcium diet pattern and lower physical activity in this research, which could suggest that the effect of dietary pattern on bone could be greater than the effect of physical activity.

Among Chinese rural and urban students, we found the results reflected rural-urban differences in the prevalence of overweight/obesity, central adiposity, and elevated blood pressure/hypertension. This may be explained by the fact that urban students followed Westernization and meat diet dietary patterns more than rural ones. A positive relationship was found between a Westerization dietary pattern and the risk of overweight/obesity and central obesity in this research. Kim et al. pointed out that in this dietary pattern, the ingested fat is more than recommended, which will increase the risk of obesity [25]. Similar results were also reported by Lutsey et al. [26] and Olser et al. [27]. However, the prevalence of obesity in children and adolescents increases the risk of suffering from cardiovascular diseases, type 2 diabetes, stroke, osteoporosis, and some cancers when they come of age [28,29,30]. We found that the meat diet pattern has a positive relationship with the risk of elevated blood pressure/hypertension in this survey. The meat dietary pattern is also an unhealthy dietary pattern. Excessive intake of meat will cause obesity [31,32], and especially red meat in the long term will increase the risk of diseases, such as elevated blood pressure and atherosclerosis [33,34,35]. The Chinese and Western dietary pattern was inversely related with the risk of low bone mineral quality in our study. This dietary pattern includes food such as milk and beans. Research shows that eating fruit, vegetables, milk, and beans often is beneficial to skeletal development [36]. Wosje et al. found that the dietary pattern of adequate vegetables and fruit is relevant to children’s high bone mass [37]. Lanou et al. believed that the dairy food consumption of children and adolescents is significantly relevant to bone health index [38]. Overall, these findings could explain the different prevalence of overweight/obesity, central adiposity, and elevated blood pressure/hypertension between rural and urban students.

There are three main limitations in this research. First of all, due to the cross-sectional design, it failed to completely confirm the causal relation between dietary patterns and adverse outcomes. Thus, the findings are expected to be verified in future research. Moreover, owing to the retrospective evaluation of dietary exposure, the research failed to totally exclude recall bias. At last, the ultrasound bone densitometer may be less accurate than the dual energy X-ray bone densitometer (DXA), which may have compromised BMD findings, but DXA scans were not suitable for an investigation of such a large scale.

## 5. Conclusions

In conclusion, rural-urban disparity in dietary patterns was found in our study, and different dietary patterns were associated with the risk of some adverse outcomes. Through the adjustment for all confounding factors, the Westernization structure could increase the risk of overweight/obesity and central adiposity, the meat structure could increase the risk of elevated blood pressure/hypertension, while the risk of low bone mineral quality could be reduced by the Chinese and Western structure. Therefore, there were different prevalences of the adverse outcomes between rural and urban students.

## Figures and Tables

**Table 1:** Factor loadings for major dietary patterns.

Food Groups	Dietary Patterns		
	Chinese and Western	Westernization	Meat Diet
Rice and Rice products	**0.669**	**0.441**	0.317
Flour and flour products (including big steamed buns, noodles, cake, etc.)	**0.658**	**0.438**	0.338
Whole grains (including corn, sorghum, oats, potato, etc.)	**0.626**	0.359	−0.009
Fresh vegetables	**0.760**	0.388	0.239
Fresh fruit	**0.639**	**0.475**	0.323
Fat meat	0.103	**0.444**	**0.738**
Beef and mutton	0.322	0.232	**0.755**
Poultry	**0.482**	0.268	**0.628**
Animal liver	0.250	0.251	**0.719**
Eggs	**0.748**	0.371	0.344
Freshwater fishes and shrimps, etc.	**0.657**	0.156	**0.504**
Deep-sea fish (including Ribbon fish, cod, sardines)	**0.552**	0.177	**0.494**
Milk and dairy products (including milk, yogurt, milk tablets, etc.)	**0.714**	0.235	0.234
Beans and bean products	**0.633**	0.220	0.398
Hamburgers and Fried foods	0.276	**0.507**	0.297
Processed products (such as bacon, salted fish, pickle)	0.246	**0.400**	**0.489**
Nuts (such as walnut, peanut, etc.)	**0.619**	0.302	0.211
Snacks (including cookies, chocolate, cakes, etc.)	**0.421**	**0.711**	0.288
Coke, Sprite	0.196	**0.789**	0.220
Coffee	0.371	**0.697**	0.258
Sugar (such as candy, sugar, brown sugar, etc.)	**0.425**	0.314	0.289
Ice cream	0.375	**0.720**	0.286
Instant noodles	0.307	**0.728**	0.235
Barbecue	**0.402**	**0.700**	0.293
Percentage of variance explained	26.352	22.217	17.126

The factor analysis with varimax rotation was used to recognize major dietary structures. Foods listed in the table were from the self-administered diet questionnaire. Values ≥ 0.40 were listed in the table in bold.

**Table 2:** Rural-urban characteristics of the study participants.

Participants	Characteristics	Rural (949)	Urban (641)	χ^2^	*p*
Age (years)	Mean ± SD	15.18 ± 1.27	14.98 ±1.16	-	-
Sex	male	448 (47.2%)	318 (49.6%)	0.884	0.347
	female	501 (52.8%)	323(50.4%)	-	-
Place for meals	canteen	548 (57.7%)	101 (15.8%)	507.891	**0.000**
	eatery	275 (29.0%)	108 (16.8%)	-	-
	at home	126 (13.3%)	432 (67.4%)	-	-
Economic status	Wealthy (The family’s per capita income > 3000)	186 (19.6%)	329 (51.3%)	213.068	**0.000**
	General (The family’s per capita income 500~3000)	325(34.2%)	202 (31.5%)	-	-
	poor (The family’s per capita income < 500)	438 (46.2%)	110(17.2%)	-	-
Education of Father	Primary school or below	216 (22.8%)	46 (7.2%)	229.139	**0.000**
	Junior High school	502 (52.9%)	202 (31.5%)	-	-
	Senior High school or above	231 (24.3%)	393 (61.3%)	-	-
Education of Mother	Primary school or below	403(42.5%)	162(25.3%)	94.776	**0.000**
	Junior High school	382 (40.3%)	238 (37.1%)	-	-
	Senior High school or above	164(17.2%)	241 (37.6%)	-	-
Passive smoking	Yes	509 (53.6%)	338 (52.7%)	0.126	0.723
	No	440(46.4%)	303 (47.3%)	-	-
Strenuous exercise	<3 times/week	493(51.9%)	343 (53.5%)	0.374	0.653
	>3 times/week	456 (48.1%)	298 (46.5%)	-	-
Medium-intensity exercise	<3 times/week	335 (35.3%)	414 (64.6%)	131.696	**0.000**
	>3 times/week	614 (64.7%)	227 (35.4%)	-	-
Low-intensity exercise	<3 times/week	312 (32.9%)	396(61.8%)	129.380	**0.000**
	>3 times/week	637 (67.1%)	245(38.2%)	-	-
Nutritious supplements	Yes	325 (34.2%)	493 (76.9%)	278.794	**0.000**
	No	624 (65.8%)	148(23.1%)	-	-

Chi-square test and associated p values are provided. Data were presented as mean ± SD, or number of cases (%).

**Table 3:** Rural-urban differences of primary and middle school students across tertiles of major dietary pattern scores.

Dietary Pattern	Rural	Urban	χ^2^	*p*
*N*	949	641	-	-
Chinese and Western	-	-	49.693	0.000
T1	382 (40.3%)	192(29.9%)	-	-
T2	325 (34.2%)	178 (27.8%)	-	-
T3	242 (25.5%)	271 (42.3%)	-	-
Westernization	-	-	36.947	0.000
T1	351 (37.0%)	162 (25.3%)	-	-
T2	352 (37.1%)	230 (35.9%)	-	-
T3	246 (25.9%)	249 (38.8%)	-	-
Meat diet	-	-	36.423	0.000
T1	412 (43.4%)	185 (28.9%)	-	-
T2	295 (31.1%)	231 (36.0%)	-	-
T3	242 (25.5%)	225 (35.1%)	-	-

Chi-square test and associated *p* values were provided. T1 the lowest tertile of dietary pattern scores; T2 the intermediate tertile of dietary pattern scores; T3 the highest tertile of dietary pattern scores.

**Table 4:** Differences of basic characteristics between urban and rural participants.

Characteristics	Boys			Girls		
	Urban	Rural	*P*	Urban	Rural	*p*
*N*	498	349	-	451	292	-
Height (m)	1.69 ± 0.58	1.67 ± 0.54	0.612	1.59 ± 0.51	1.57 ± 0.49	0.596
Weight (kg)	62.14 ± 9.21	60.22 ± 11.34	<0.01	52.39 ± 5.03	51.41 ± 6.29	<0.05
Waist circumference (cm)	72.12 ± 8.88	68.92 ± 7.69	<0.01	66.54 ± 7.63	64.74 ± 6.86	<0.01
Hip circumference (cm)	88.95 ± 7.49	85.52 ± 6.61	<0.01	88.50 ± 8.15	86.08 ± 5.02	<0.01
Systolic blood pressure (mmHg)	116.71 ± 11.41	115.84 ± 11.07	0.269	108.89 ± 10.27	108.41 ± 9.12	0.516
Diastolic blood pressure (mmHg)	64.27 ± 8.50	65.03 ± 10.00	0.234	62.77 ± 7.02	63.02 ± 7.11	0.637
Speed of sound (m/s)	1561 ± 28.63	1531 ± 31.99	<0.01	1541 ± 26.94	1529 ± 26.32	<0.01
Body mass index distribution (kg/m^2^)	-	-	<0.05	-	-	<0.05
wasting	56 (11.2%)	54 (15.5%)	-	98 (21.7%)	50 (17.1%)	-
normal weight	304 (61.0%)	222 (63.6%)	-	235 (52.1%)	179 (61.3%)	-
overweight/obesity	138 (27.8%)	73 (20.9%)	-	118 (26.2%)	63 (21.6%)	-
Central adiposity	-	-	<0.05	-	-	<0.05
Yes	141 (28.3%)	77 (22.1%)	-	115 (25.5%)	55 (18.8%)	-
No	357 (71.7%)	272 (77.9%)	-	336 (74.5%)	237 (81.2%)	-
Blood pressure distribution (mmHg)	-	-	0.496	-	-	0.910
normal blood pressure	432 (86.7%)	297 (85.1%)	-	389 (86.3%)	251 (86.0%)	-
elevated blood pressure/hypertension	66 (13.3%)	52 (14.9%)	-	62 (13.7%)	41 (14.0%)	-
Bone mineral quality distribution (*Z* value)	-	-	<0.01	-	-	<0.01
Normal	476 (95.6%)	303 (86.8%)	-	418 (92.7%)	248 (84.9%)	-
Low bone quality	22 (4.4%)	46 (13.2%)	-	33 (7.3%)	44 (15.1%)	-

Data were presented as mean ± SD, or number of cases (%). The associations between categorically demographic variables were assessed using χ^2^ analysis. *T*-test was used to compare continuous variables (mean ± SD).

**Table 5:** Odds ratios (95% confidence intervals) for components of adverse outcome across tertiles of major dietary pattern scores.

Dietary Patterns in Models	Overweight/Obesity	Central Adiposity	Elevated Blood Pressure/Hypertension	Low Bone Quality
	OR ^#^ (95% CI)	OR ^#^ (95% CI)	OR ^#^ (95% CI)	OR ^#^ (95% CI)
Model 1				
Westernization				
T2	1.484 (1.031–2.045) *	2.587 (1.199–5.859) *	1.085 (0.779–1.445)	1.033(0.795–1.383)
T3	1.769 (1.153–2.736) *	4.801 (1.848–12.307) *	1.114 (0.825–1.674)	0.955 (0.829–1.101)
Meat diet				
T2	1.238 (1.051–1.882) *	1.218 (1.004–1.477) *	1.318 (1.006–1.930) *	0.972 (0.729–1.348)
T3	1.457 (1.001–2.143) *	1.229 (1.043–2.903) *	1.436 (1.139–1.810) *	0.918 (0.684–1.233)
Chinese and Western				
T2	0.932 (0.650–1.337)	0.907 (0.582–1.411)	0.618 (0.253–0.932) *	0.974 (0.269–3.520)
T3	0.894 (0.620–1.290)	1.065 (0.716–1.584)	0.479 (0.218–0.831) *	1.097 (0.613–1.965)
Model 2				
Westernization				
T2	1.386 (1.149–2.034) *	2.785 (1.278–6.143) *	0.913 (0.676–1.232)	1.192 (0.855–1.663)
T3	1.769 (1.146–2.731) *	5.191 (1.998–13.403) *	0.778 (0.579–1.045)	1.224 (1.030–1.771) *
Meat diet		-	-	-
T2	1.154(0.752–1.771)	1.566 (1.230–3.349) *	1.421(1.098–1.876) *	0.911 (0.620–0.255)
T3	1.232 (1.021-1.521) *	1.775 (1.172–3.591) *	1.678 (1.134–2.135) *	0.920 (0.626–1.354)
Chinese and Western				
T2	0.952 (0.627–1.447)	0.834 (0.693–0.998) *	1.114 (0.773–1.606)	0.621 (0.512–0.832) *
T3	1.073 (0.697–1.652)	0.712(0.445–0.867) *	0.956 (0.712–1.283)	0.558 (0.414–0.901) *
Model 3				
Westernization				
T2	1.370 (1.087–2.048) *	2.391 (1.173–4.872) *	1.078 (0.892–1.301)	0.942 (0.736–1.207)
T3	1.925 (1.299–2.852) *	5.511 (2.222–13.667) *	1.179 (0.770–1.804)	0.980 (0.788–1.217)
Meat diet				
T2	0.938 (0.745–1.180)	0.964 (0.766–1.213)	1.477 (1.087–2.006) *	1.084 (0.893–1.315)
T3	1.113 (0.819–1.512)	1.051 (0.892–1.331)	1.576 (1.156–2.147) *	1.068 (0.761–1.498)
Chinese and Western				
T2	0.961 (0.630–1.446)	1.019 (0.844–1.229)	0.983 (0.811–1.191)	0.603 (0.453–0.898) *
T3	1.088 (0.702–1.684)	0.990 (0.711–1.380)	1.135 (0.776–1.648)	0.421(0.289–0.559) *

Multivariable logistic regression was applied to recognize the risk of overweight/obesity, central adiposity, abnormal blood pressure, and low bone quality for each main dietary structure. ^#^ OR is the odds ratio of T2 or T3 vs. T1, and T1 is regarded as reference group; * Significantly different from T1: * *p* < 0.05; T1: the lowest tertile of dietary pattern score; T2: the intermediate tertile of dietary pattern score; T3: the highest tertile of dietary pattern’s score; Model 1: unadjusted; Model 2: adjusted for sex, passive smoking, drinking, calcium supplements. SOS (speed of sound) was further adjusted for BMI (body mass index); Model 3: physical activity was further adjusted.
